# Immune-modulatory genomic properties differentiate gut microbiota of infants with and without eczema

**DOI:** 10.1371/journal.pone.0184955

**Published:** 2017-10-19

**Authors:** Seungdae Oh, Gaik Chin Yap, Pei-Ying Hong, Chiung-Hui Huang, Marion M. Aw, Lynette Pei-Chi Shek, Wen-Tso Liu, Bee Wah Lee

**Affiliations:** 1 Department of Civil Engineering, Kyung Hee University, Yongin-si, Gyeonggi-do, Republic of Korea; 2 Department of Civil and Environmental Engineering, University of Illinois at Urbana-Champaign, Urbana, IL, United States of America; 3 Department of Paediatrics, Yong Loo Lin School of Medicine, National University of Singapore, Singapore, Singapore; 4 Division of Biological and Environmental Science and Engineering, King Abdullah University of Science and Technology, Thuwal, Saudi Arabia; 5 Water Desalination and Reuse Center, Division of Biological and Environmental Science and Engineering, King Abdullah University of Science and Technology, Thuwal, Saudi Arabia; 6 Department of Paediatrics, Khoo Teck Puat-National University Children's Medical Institute, National University Health System, Singapore, Singapore; University of Georgia, UNITED STATES

## Abstract

Gut microbiota play an important role in human immunological processes, potentially affecting allergic diseases such as eczema. The diversity and structure of gut microbiota in infants with eczema have been previously documented. This study aims to evaluate by comparative metagenomics differences in genetic content in gut microbiota of infants with eczema and their matched controls. Stools were collected at the age of one month old from twelve infants from an at risk birth cohort in a case control manner. Clinical follow up for atopic outcomes were carried out at the age of 12 and 24 months. Microbial genomic DNA were extracted from stool samples and used for shotgun sequencing. Comparative metagenomic analysis showed that immune-regulatory TCAAGCTTGA motifs were significantly enriched in the six healthy controls (C) communities compared to the six eczema subjects (E), with many encoded by *Bifidobacterium* (38% of the total motifs in the C communities). Draft genomes of five *Bifidobacterium* species populations (*B*. *longum*, *B*. *bifidum*, *B*. *breve*, *B*. *dentium*, and *B*. *pseudocatenulatum*) were recovered from metagenomic datasets. The *B*. *longum* BFN-121-2 genome encoded more TCAAGCTTGA motifs (4.2 copies per one million genome sequence) than other *Bifidobacterium* genomes. Additionally, the communities in the stool of controls (C) were also significantly enriched in functions associated with tetrapyrrole biosynthesis compared to those of eczema (E). Our results show distinct immune-modulatory genomic properties of gut microbiota in infants associated with eczema and provide new insights into potential role of gut microbiota in affecting human immune homeostasis.

## Introduction

Eczema, a complex inflammatory disease, is one of the earliest manifestations of the allergic march. It is also the most common chronic childhood skin disease affecting up to 20% of children [1]. Emerging evidence showing gut microbiota is important for the normal development of immune system, an extension of the hygiene hypothesis, the ‘microflora hypothesis’ of allergic diseases highlights the potential role of gut microbiota in modulating host immunity has been proposed [2].This hypothesis is supported by some population studies showing that reduced microbial diversity in early life is associated with the development of eczema [3–5], although others have failed to support this finding [6–8].

Through the evaluation of microbial 16SrRNA genes, studies have shown that the composition of the fecal microbiota community differs between eczema and healthy controls in infancy. For instance, our and other previous studies have shown that eczema was significantly associated with higher abundance of *Enterocococcus* species, *E*. *coli* and *Clostridium difficile* and lower abundance of *Bifidobacterium* [3, 9–13]. Recent studies demonstrated that metabolites of gut microbiota, such as the short-chain fatty acids, could promote the generation of peripheral regulatory T cells and protect against allergic inflammation [14–16]. Decreased levels of butyrate and propionate in stools of subjects with eczema and this association has been shown to be specifically related to the intraspecies compositional change in gut *Faecalibacterium prausnitzii* leading to a reduction in the number of high butyrate producers [17].

A previous study reports significant differences in fecal microbial community diversity between healthy infants and those with eczema even at very early stages (one to four months of age) [18]. In the present study, we performed metagenomic analysis of stool samples collected at one month old to explore the changes of gut microbiome in relation to the development of eczema. Our results show differential immune-modulatory genomic potential that distinguishes gut microbiota of healthy infants from those with eczema in the early life.

## Material and methods

### Infant subjects

At risk infants for atopy (at least one first degree relative with eczema, allergic rhinitis and/or asthma) were recruited to participate in a randomized double-blind trial that aimed to determine the protective effects of infant cow’s milk formula supplemented with probiotics in early life on allergic outcomes (ClinicalTrials.gov Identifier: NCT00318695) [19]. Of the 30 infants who developed eczema in the first two years of life in the placebo arm of the study, those with the more severe eczema (highest SCORAD) and available stools at one month of age were examined. The stool samples examined in this study were collected at one month from 12 infants of the placebo arm of a birth cohort of at-risk infants. Collection and processing of stool samples were carried out as described previously [20]. The time point of one month was chosen as a relatively early time point in infancy before the outcome of early onset eczema occurs.

Six infants who developed eczema in the first two years were matched with their healthy controls for the following confounding factors: age, mode of delivery and feeding at one month. All healthy controls did not have eczema, asthma, rhinitis in the first two years and had negative skin prick tests (SPT) to cow’s milk, egg white, fish, soy protein, peanut, *Dermatophagoides pteronyssinus* and *Blomia tropicalis* when examined at the age of 12 and 24 months. Severity of eczema was assessed by SCORAD. Written informed consent was obtained from all families. The study was approved by National Healthcare Group’s Domain Specific Review Board (DSRB) (Ref Code: 2011/02132).

### DNA extraction and metagenome sequencing

Stools samples stored in TN (10 mM Tris-HCl [pH = 8], 150 mM NaCl) buffer as described previously [20] were used to extract microbial genomic DNA by UltraClean soil DNA isolation kit with modifications (MO BIO Laboratories, Carlsbad, CA, USA). Briefly, one milliliter of TN stool sample was centrifuged for 30s at 10,000 × g. TN buffer was removed. The content from the 2ml Bead Solution Tube of the UltraClean soil DNA isolation kit was added into the fecal pellet and followed by adding 12μl of lysozyme (1000mg/ml) and 12μl of achromopeptidase (1mg/ml). The sample was vortexed and incubated at 37°C for one hour. After that, 60 μl of solution S1 were added and DNA was extracted following the manufacturer’s instructions. The DNA samples were sent to the Roy J. Carver Biotechnology Center, University of Illinois, Urbana for shotgun sequencing. Roche GS Rapid library preparation (Roche, Basel, Switzerland) and Nextera library preparation (Illumina, San Diego, CA, USA) were carried out for Roche 454 and Illumina shotgun sequencing respectively.

### Sequence preprocessing and assembly

Metagenomic datasets sequenced using different sequencing technologies (e.g., Illumina HighSeq 2500, MiSeq V2, and Roche 454 FLX Titanium) were combined into 12 metagenomic datasets by 12 sample sources, respectively. Metagenomic reads were trimmed using a Q = 13 Phred quality score cutoff using SolexaQA2 [21]. Metagenomic reads were searched against a human genome sequence (accession no. AC_000142) using BLAST with a cutoff of > 90% nucleotide identity and > 50 matched length. The aligned metagenomic reads (< 1% of the total) were discarded and the remaining metagenomic reads were used for further analysis. The hybrid protocol that employs a combination of Velvet, SOAPdenovo2, and Newbler 2.8 assemblers was used to assemble each metagenomic dataset, as described previously [22–25].

### Phylogenetic and functional annotation of metagenomic sequences

Metagenomic reads obtained from HighSeq 2500 and MiSeq V2sequencing were searched against the small subunit ribosomal RNA (SSU rRNA) gene (hyper variable V9 region: positions 1380–1510) database [26] using BLASTn with a cutoff of > 80% nucleotide identity and > 90% target length coverage. About 7,000 to 14,000 metagenomics reads encoding the V9 region of 16S rRNA genes were retrieved from 12 datasets, respectively. The V9 region-encoding sequences were preprocessed and analyzed for constructing rarefaction curves estimating Shannon diversity and Chao diversity indices, using the MOTHUR package [27]. Sequences were rarefied to 7,000 sequences per sample to estimate the alpha diversity indices. Protein-coding genes on assembled contigs were predicted using MetaGeneMark [28]. To examine the phylogenetic affiliation of the protein-coding genes, the amino acid sequences were searched against all bacterial and archaeal genome sequences available in the GenBank database (ftp://ftp.ncbi.nih.gov/, as of January 2014), using BLASTp with a cut-off of > 40% identity and > 50% query length coverage. To functionally annotate the gene products, the amino acid sequences were searched against the SEED subsystems protein database [29] using BLASTp with a cutoff of > 40% amino acid sequence identity and > 50% query length coverage for a match.

To estimate the relative abundance (expressed as coverage) of a gene or genome sequence in a metagenomic dataset, metagenomic reads obtained from HighSeq 2500 and MiSeq V2 sequencing were mapped on the sequence with > 95% identity and > 50% query length coverage. The length of all mapped metagenomic reads on the sequence was summed and divided by the length of the sequence. The value was subsequently normalized by the size of the metagenomic dataset (i.e. normalized to 10 Gb of metagenomic sequences), which provided a normalized relative abundance of the sequence across metagenomic datasets. For estimating relative abundance (expressed as percentage) of phylogenetic groups and metabolic pathways, the number of metagenomic reads mapped on the protein-coding genes (phylogenetically and functionally annotated) was also considered for the normalization.

### Genome reconstruction of *Bifidobacterium* populations

To reconstruct draft genome sequences of *Bifidobacterium* populations, binning of the assembled contigs of individual metagenomic datasets were first carried out based on metagenomic read coverage, tetranucleotide frequency, and the occurrence of unique marker genes using MaxBin [30]. Individual genome bins obtained using MaxBin were searched against all sequenced *Bifiidobacterium* genomes in GenBank using BLASTn. Among all genome bins clustered, best-match analysis screened six genome bins showing > 95% average genome identity with the *Bifidobacterium* genomes in GenBank. To reduce possible mis-assigned contigs (e.g., false positives) within the six genome bins, contigs that showed less than 95% nucleotide identity to the *Bifidobacterium* genomes in GenBank were excluded.

### Statistical analysis

Statistical tests for differentially abundant features (e.g., microbial taxa, functional profiles, and genome coverage) on the basis of normalized count data were performed using Metastats [31]. Metastats utilizes a non-parametric t-test [32] and separately employs a Fisher’s exact test for rare features, performing well for comparing metagenomics sequencing data. The *P* values were computed with 1000 permutations using Metastats and the significant threshold was set at *P* < 0.05.

### Nucleotide sequence accession numbers

The metagenomic datasets used in this study were deposited in GenBank under the accession numbers: SRR1793416 (subject ID:121), SRR1825367 (ID: 128), SRR1825362 (ID: 141), SRR1779472 (ID: 157), SRR1789035 (ID: 161), SRR1818241 (ID: 165), SRR1819806 (ID: 166), SRR1822318 (ID: 170), SRR1783777 (ID: 176), SRR1821190 (ID: 177), SRR1799892 (ID: 192), and SRR1793377 (ID: 221) from the Illumina Hiseq 2500 sequencer, SRR1793408 (ID: 121), SRR2155554 (ID: 128), SRR1822319 (ID: 141), SRR1777715 (ID: 157), SRR1791585 (ID: 161), SRR1818227 (ID: 165), SRR1802723 (ID: 166), SRR1818235 (ID: 170), SRR1785909 (ID: 176), SRR1818231 (ID: 177), SRR1793410 (ID: 192), SRR1793359 (ID: 221) from the Miseq V2 sequencer, and SRR1763038 (ID: 157), SRR1766383 (ID: 157), SRR1773183 (ID: 161), SRR1768458 (ID: 176), and SRR1776761 (ID: 221) from the Roche 454 FLX Titanium sequencer.

## Results

### Clinical characteristics

The clinical characteristics of six eczema children (E group) (subject ID: 161, 221, 128, 141, 170, 177) by two years of age (two caesarean delivered and exclusively formula fed children, four vaginal delivered and partial formula fed children), and their matched healthy controls (C group) are shown in Table 1. Three children developed eczema by 1 month of age, two children by 3 months and one child by 12 months. The severity of eczema was assessed by SCORAD and was determined to range from 7.4 to 35.9 at different time points within the first two years of age. None of the children had positive skin prick test arising from dietary and inhalant allergens at 12 months. At the two year follow up, three children with eczema were sensitized to *D*. *pteronyssinus*, and one to *B*. *tropicalis*. For dietary allergens, one eczema child was sensitized to peanut, and another sensitized to egg white. Rhinitis and asthma were subsequently diagnosed in two eczema children separately. Up till the time of the one month stool sampling, all subjects had not received antibiotics.

**Table 1:** Clinical characteristics of infants.

Subject	Ethnicity	Mode of delivery	Feeding at one month	Skin Prick Test at two years	allergic manifestations by two years	SCORAD (month of diagnosis)	Family allergic history
157	Malay	LSCS	Total formula	Negative	Nil	N.A.	Mother
176	Indian	LSCS	Total formula	Negative	Nil	N.A.	Father
121	Malay	Vaginal delivery	Breastfeeding and formula	Negative	Nil	N.A.	Mother
165	Malay	Vaginal delivery	Breastfeeding and formula	Negative	Nil	N.A.	Mother
166	Malay	Vaginal delivery	Breastfeeding and formula	Negative	Nil	N.A.	Father
192	Indian	Vaginal delivery	Breastfeeding and formula	Negative	Nil	N.A.	Mother
161	Malay	LSCS	Total formula	Positive for *Der p*	Eczema	7.4 (3 months)	Father
221	Chinese	LSCS	Total formula	Negative	Eczema	21.2 (1 month), 11.6 (6 months)	Mother
128	Malay	Vaginal delivery	Breastfeeding and formula	Positive for *Blo t*, *Der p* and peanut	Eczema	22.4 (1 month), 27 (3 months), 11.3 (12 months), 20.1 (24 months)	Father, Mother
141	Chinese	Vaginal delivery	Breastfeeding and formula	Positive for egg white	Eczema, Rhinitis	21.2 (1 month), 21.2 (6 months), 20.9 (12 months), 7.6 (24 months)	Father
170	Malay	Vaginal delivery	Breastfeeding and formula	Negative	Eczema	21.4 (3 months), 10.6 (6 months)	Mother
177	Malay	Vaginal delivery	Breastfeeding and formula	Positive for *Der p*	Eczema, Asthma	24.8 (12 months), 35.9 (24 months)	Father

LSCS, Lower segment caesarean section; Der p, *Dermatophagoides pteronyssinus*; Blo t, *Blomia tropicalis*.

### Community phylogenetic structure and diversity

**Fig 1:**
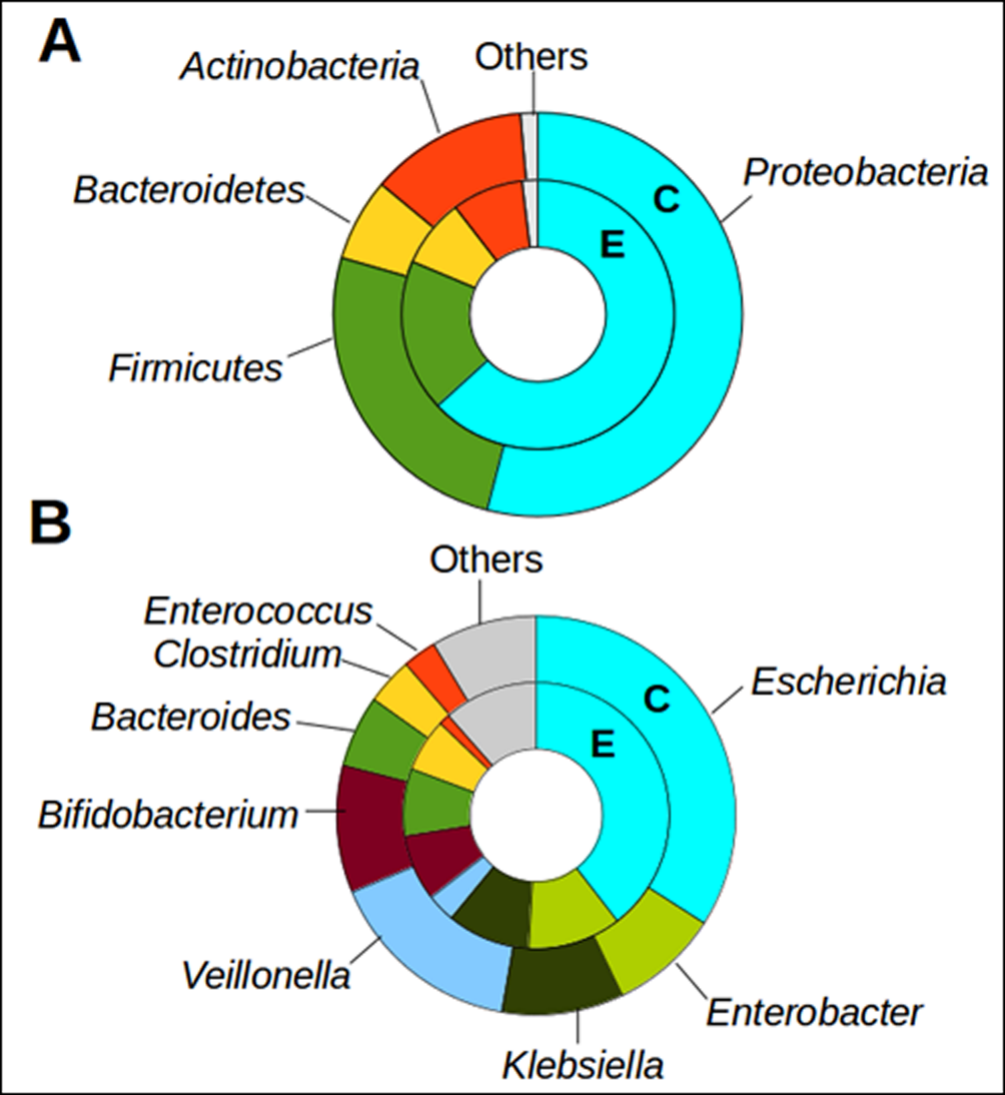
Phylogenetic composition of microbial communities. Average relative abundance of major phyla (Avg. > 5% of the total community) **(A)** and genera (Avg. > 1%) **(B)** of C (outer circle) and E (inner circles) communities. ‘Others’ represents the combined fraction of the remaining minor taxa.

The community phylogenetic structure of the 12 microbial communities (six E and six C communities) was analyzed using both metagenomic and 16S rRNA gene sequences reads. Phylogenetic analysis of metagenomic sequences revealed that four phyla dominated both C and E communities: *Proteobacteria* (54% and 63% for C and E, respectively), *Firmicutes* (26% and 18%), *Actinobacteria* (13% and 8%), *Bacteroidetes* (7% and 8%) (Fig 1A). This observation was congruent with that based on 16S rRNA gene sequences (S1 Fig). The phylogenetic analysis of metagenomic sequences also revealed abundances of *Escherichia* (33% and 38%), followed by *Veillonella* (15% and 3%), *Bifidobacterium* (10% and 8%), *Klebsiella* (9% and 10%), *Enterobacter* (8% and 11%), *Bacteroides* (6% and 8%), *Clostridium* (4% and 6%), and *Enterococcus* (3% and 1%) (Fig 1B). None of the major phyla and genera showed significant difference in relative abundance between C and E communities. *Veillonella* exhibited five times higher relative abundance, on average, in C communities; however, it was not significantly different (*P* = 0.13 by Metatstats) due to a significant variation of the abundances within each community group. Rarefaction curves showed that the number of OTU (i.e., defined at the 97% sequence identity level) sequences almost saturated the OTU diversity within individual metagenomic datasets (S2 Fig). The Shannon diversity and the Chao richness indices estimated based on the OTU sequences showed 2.3 ± 0.5 and 87 ± 32 for C communities, respectively, which were comparable to 2.1 ± 0.7 and 91 ± 24 for E communities (S1 Table). The Chao richness estimates (39–123) for C and E communities were comparable to the number of OTUs (130 ± 39) previously observed in infant gut microbial communities using 16S rRNA gene pyrosequencing [9]. Our results revealed no significant difference (*P* > 0.8) in OTU diversity and richness between C and E communities.

Full-length bacterial 16S rRNA gene sequences contain at least nine hypervariable regions (V1-V9), each of which can have a relatively higher resolution for specific bacterial taxa. V3 and V6 regions are often used and resulted in phylogenetic profiling results of microbial communities similar to those using full-length sequences [33]. Hence, we retrieved metagenomic reads encoding V3 (positions 338–533) [25] using the same method as described for the V9 region analysis. Only MiSeq metagenomic reads were considered for this analysis, since the reads (250 bp-long) could fully cover the V3 region. The analysis could identify 1,600 to 4,400 V3-encoding metagenomic reads from each dataset, respectively. Both Shannon diversity and Chao richness indices estimated using 1,600 sequences (i.e., rarefied to the lowest number of sequences retrieved) did not differ significantly between C (2.2 ± 0.4 and 95 ± 42, respectively) and E communities (2.1 ± 0.3 and 86 ± 40, respectively), consistent with the results obtained using the V9 region.

### Differential abundance of immunomodulatory motifs

**Fig 2:**
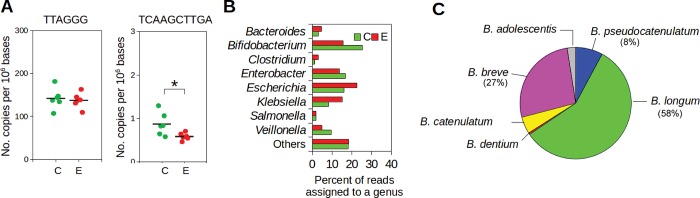
Immunosuppressive motifs differentiate C communities from E communities. (A) Frequencies of two immunosuppressive motifs (TTAGGG and TCAAGCTTGA) in C and E communities. The asterisk denotes *P* < 0.05 by Metastats. (B) Phylogenetic affiliation of the metagenomic reads encoding the TCAGCTTGA motifs in C and E communities, respectively. (C) Phylogenetic affiliation of the *Bifidobacterium* TCAGCTTGA motifs observed in C communities to available *Bifidobacterium* species genomes (GenBank).

Unmethylated cytosine phosphate guanine (CpG) dinucleotides motifs are overrepresented in bacteria genomes compared to vertebrate ones [34]. The CpG motifs can act as immunostimulants that induce human immune responses through Toll like receptor 9 (TLR-9) [35]. To compare the immune-stimulatory genomic potential between C and E communities, metagenomic reads were searched against five CpG motifs whose immunological activities were reproducibly observed previously (S2 Table). The mean fold changes (C vs E communities) of the CpG motifs occurrence ranged from 0.95 to 1.1. Immune-suppressive motifs (TTAGGG and TCAAGCTTGA) modulate human immune responses by counteracting the effects of the immune-stimulatory CpG motifs [36]. While the occurrence of the TTAGGG motifs remained comparable between C and E communities, the TCAAGCTTGA motifs appeared significantly more abundant (1.5 mean fold change with *P* < 0.05 by Mann-Whitney U test) in C communities (Fig 2A). The phylogenetic affiliation of the metagenomic reads encoding the TCAAGCTTGA motifs were examined by searching the metagenomic reads against all available archaeal and bacterial genomes, using BLASTx with a cutoff of > 80% amino acid sequence identity and > 50% query length coverage. The analysis revealed that genomes of the eight major genera listed in Fig 1B accounted for > 80% of the total TCAAGCTTGA motifs in both C and E metagenomic datasets. Notably, the TCAAGCTTGA motifs were particularly enriched in *Bifidobacterium* (25% and 16% in C and E metagenomic datasets, respectively), followed by *Enterobacter* (17% and 14%), *Escherichia* (16% and 23%), and *Veillonela* (9% and 5%) (Fig 2B). Considering that *Bifidobacterium* contributed to only 8–10% phylogenetic relative abundances, the substantial contribution of *Bifidobactrium* (25% and 16%) to the total TCAAGCTTGA motifs suggested that the *Bifidobacterium* in the neonatal gut microbiota encoded more copies of the motifs in their genomes than those of other major genera.

To ascertain whether the particular enrichment of the TCAAGCTTGA motifs in the *Bifidobacterium* observed in the present study is the general genomic property in *Bifidobacterium*, we analyzed the TCAAGCTTGA motifs in all complete genomes of the major eight genera (listed in Fig 1B) which were collected from GenBank (S3 Fig). While the average occurrence (per 10^6^ bp) was 1.7 in *Bifidobacterium* genomes, other genera genomes showed less than 1.0. The analysis of complete isolate genomes publicly available corroborated the higher occurrence of the TCAAGCTTGA motifs in the *Bifidobacterium* genus. The phylogenetic affiliation of the metagenomic reads encoding TCAAGCTTGA motifs that were assigned to *Bifidobacterium* was examined further. This showed that the majority of the TCAAGCTTGA motifs were phylogenetically related to *B*. *longum* (58% of the total *Bifidobacterium* TCAAGCTTGA motifs), followed by *B*. *breve* (27%) and *B*. *pseudocatenulatum* (8%) (Fig 2C). Taken all together, our results suggested that the level of the TCAAGCTTGA motifs significantly differed between C and E communities. Further, the TCAAGCTTGA motifs were not evenly distributed across diverse gut microbiota but enriched in specific taxa (e.g., *Bifidobacterium* and, in particular, *B*. *longum*).

### Genome construction and analysis of *Bifidobacterium* populations

Our binning approach recovered near-complete (> 90% genome completeness) draft genome sequences of *Bifidobacterium* populations. The draft genome sequences were assembled with at least 95% overlap identity. The metagenomic read recruitment on the genome sequences was carried out using HiSeq and MiSeq metagenomic reads. The analysis revealed that all draft genomes exhibit at least 22× genome coverage with 100% sequence identity. Further, the nucleotide identity of the reads mapped to the six genomes was > 98% on average, suggesting that each draft genome sequence constructed represents that of a species-like population with about 2% intrapopulation genetic variation. The genomic features of the draft genome sequences are described in S3 Table. A phylogenetic tree was constructed using DNA polymerase I genes recovered from draft genome sequences (S4 Fig). The phylogenetic relationship was congruent with the results based on average nucleotide identity (S3 Table).

**Fig 3:**
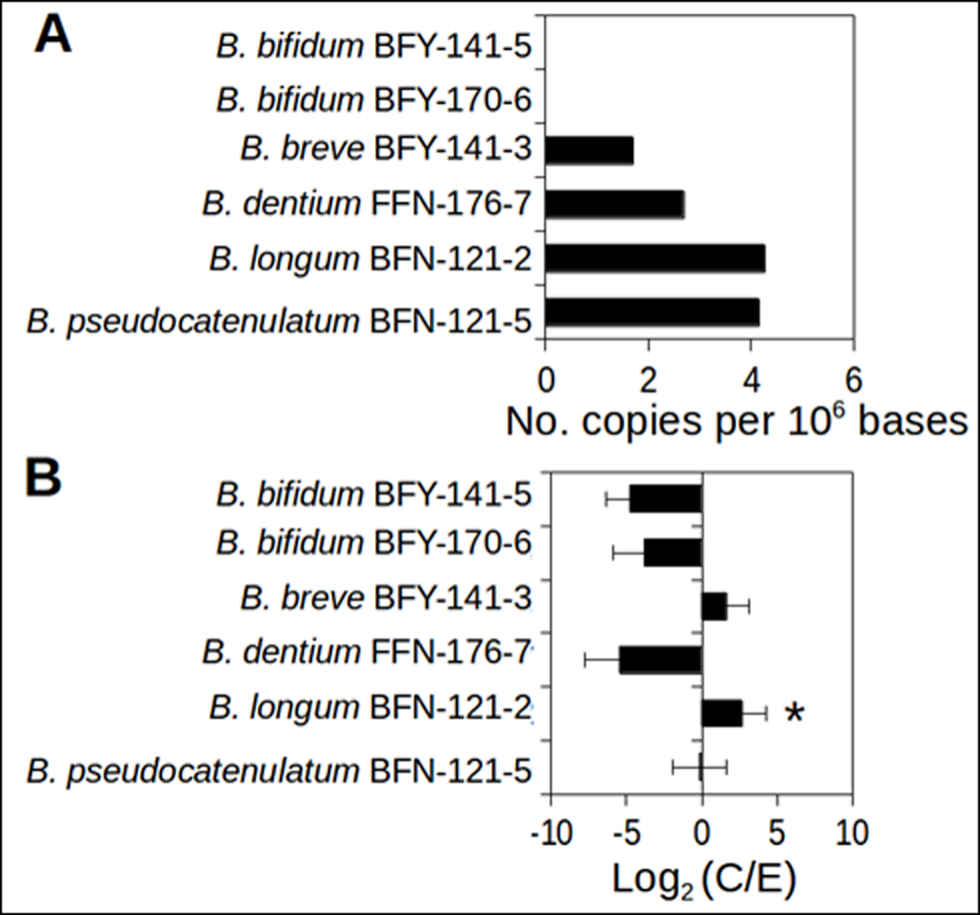
Immunosuppressive motif occurrence and relative abundance of *Bifidobacterium* sp. draft genomes. (A) Occurrence of TCAAGCTTGA motifs per 10^6^ bases. (B) Fold changes of average relative abundance of individual populations. The genome coverage was estimated for the relative abundance of a population. The bar represents the mean fold change (logarithmic scale) and the error bar represents one standard deviation from the mean. *P < 0.05 by Metastats.

The occurrence of the TCAAGCTTGA motifs in individual *Bifidobacterium* genomes was estimated. They were highly variable within the genus with results showing, 0 (copies per 10^6^ genome sequence) for *B*. *bifidum* BFY-141-5 and BFY-170-6, 1.7 for *B*. *breve* BFY-141-3, 2.7 for *B*. *dentium* FFN-176-7; 4.3 for *B*. *longum* BFN-121-2, and 4.1 for *B*. *pseudocatenulatum* BFN-121-5 (Fig 3A). Notably, the TCAAGCTTGA motifs were particularly overrepresented (> 4 copies per 10^6^ bp) in two populations: *B*. *longum* and *B*. *pseudocatenulatum*, and were four times higher than the average (1.0) of both C and E communities. We next examined the relative abundance (as genome coverage) of the six populations (Fig 3B). *B*. *longum* population was found to be significantly overrepresented (six times higher with *P* < 0.05 by Metastats) in C communities compared to E communities. While the genome coverage of *B*. *pseudocatenulatum* population was comparable between C and E communities, *B*. *bifidum* and *B*. *dentium* populations were underrepresented in C communities (Fig 3B).

### Shifts in community metabolic potential

**Fig 4:**
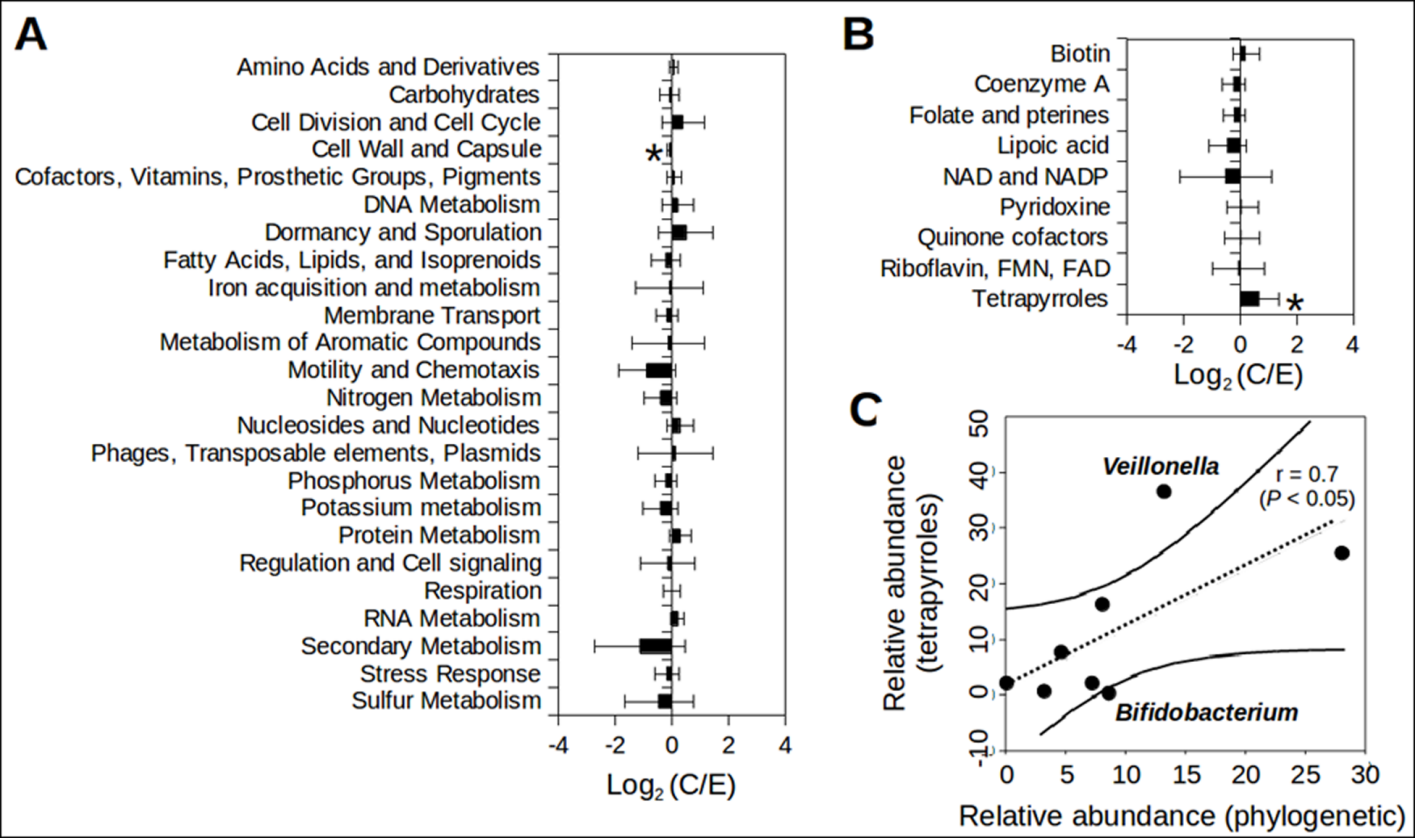
Metabolic pathways differentially abundant between C and E communities. Fold changes of average relative gene abundance assigned to 24 major metabolic categories (A) and the sub-categories related to ‘Cofactors, Vitamins, Prosthetic groups, Pigment’ (B). The bar represents the mean fold change (logarithmic scale) and the error bar represents one standard deviation from the mean. *P < 0.05 by Metastats. (C) Phylogenetic affiliation of the ‘tetrapyrrole’ biosynthesis genes in C communities. The genes related to ‘tetrapyrrole’ were taxonomically classified at genus level based on the best BLASTp search of the amino acid sequences against all available genome sequences in GenBank. The graph shows the relative abundances of the eight major genera (same as in Fig 1B) (x-axis) and the relative abundance of the tetrapyrrole-associated genes encoded by genomes of the eight major genera (y-axis). A Jarque-Bera test did not reject (P > 0.05) the null hypothesis that the ratios (y/x) of the eight genera are normally distributed. The dashed line represents ordinary least squares (OLS) regression, which shows positive correlation (Pearson correlation = 0.7 with *P* < 0.05). The solid lines represent 95% confidence bands for the OLS slope. The OLS regression analysis was performed using PAST [37]. Note that genomes of *Veilonella* and *Bifidobacterium* encoded significantly more or less tetrapyrrole-associated genes than other major genera (i.e., plotted out of the 95% confidence bands).

To further evaluate the functional potential between the subject groups, functional annotation of protein-coding genes based on best-match analysis against the SEED subsystems protein database revealed that E communities are characterized by a higher abundance (6.7% and 7.2% for C and E groups, respectively) of cell wall and capsule metabolism among the 24 major functional categories (Fig 4A). Analysis of the lower level functional categories in the cell wall and capsule metabolism did not reveal significant differences between clinical groups. C communities were significantly enriched (1.3% and 0.8% for C and E communities, respectively) in functions associated with tetrapyrrole biosynthesis (Fig 4B), the sub-category of the "Cofactors, Vitamins, Prosthetic groups, Pigment" (Fig 4A). To examine the phylogenetic affiliation of genes encoding tetrapyrrole biosynthesis in C communities, a best match BLASTp search of the amino acid sequences against all available archaeal and bacterial genomes was performed. More than 70% of the total tetrapyrrole biosynthesis genes in C communities were encoded by the eight major genera shown in Fig 1. The ordinary least squares regression (OLSR) analysis showed that the relative abundance of each genus is positively correlated with the relative abundance of the tetrapyrrole biosynthesis genes encoded by each genus (Fig 4C). Particularly, *Veillonella* appeared to have a higher number of tetrapyrrole biosynthesis-associated genes in their genomes than the expected based on its relative abundance.

## Discussion

This study investigated and compared the metagenome of infant stools at 1 month of age. Our data on functional gene categories in metabolic pathways show comparable abundance for the first 3 ranked major metabolic categories (amino acid derivatives, carbohydrates, cell division and cell cycle) to healthy infants in the same age group [38] but we are unable to make any further direct comparison. Thus, indicating comparable abundances of these fecal microbial genes between populations of infants.

Several studies have shown an association of reduced abundance of *Bifidobacterium* in the gut microbiota of infants with eczema [11, 39, 40], although these findings are not consistent between studies [4, 41–43]. Significant heterogeneity on the beneficial health effects of *Bifidobacterium* might be attributable, at least in part, to differences in *Bifidobacterium* strains. Physiological traits (e.g., adherence to intestinal epithelia) and immunomodulatory properties (e.g., induction and balance of Th1/Th2) exerted by *Bifidobacterium* are strain-dependent [44].

Knowing that the genus *Bifidobacterium* encompasses at least more than 30 taxa based on 16S rRNA gene sequence analysis [45], we evaluated various *Bifidobacterium* populations present in the infant gut communities by constructing near-complete genome sequences. The genomes constructed represent the Bifidobacteria species-like populations that have about 2% intra-population genomic variation. By examining differential abundances of the six individual populations as well as the total *Bifidobacterium* genus, we showed that the level of the specific *Bifidobacterium* population (*B*. *longum*–BFN-121-2) could discriminate C communities from E communities. However, when excluding caesarean-delivered infant samples (i.e., two in both C and E communities, respectively), although *B*. *longum*–BFN-121-2 remained four times more overrepresented in the four vaginal-delivered C communities, the *P* value (0.09) did not reach the significance level. A previous study reported a similar observation that the distribution patterns of *B*. *longum* relative to eczema are significantly affected by mode of delivery and feeding [9]. *B*. *breve* as well as *B*. *longum* is considered as an infant-type species as opposed to adult-type ones (e.g., *B*. *adolescentis*) and often associated with non-atopic infants. Our results showed that although the *B*. *breve* population was overrepresented in C communities, it did not reach statistical significance (*P* > 0.05).

Earlier studies showed that bacterial lysate, cell wall fragments, and genomic DNA of *B*. *longum* could suppress Th2-related cytokines/chemokines production [46–48]. Strains of *B*. *longum* and *B*. *infantis*, have been shown to enhance IL-10 production and induce Foxp3 T regulatory cells *in vivo*, probably via the interaction with toll like receptors (TLR) 2 [49, 50]. Besides, the genomic DNA and an immunostimulatory DNA sequence of *B*. *longum* inhibited IgE production via the interaction with TLR9 *in vitro* [47, 48, 51]. Further, a novel class of immunosuppressive motifs that counteracted CpG-mediated immune stimulation *in vitro* and *in vivo* was originally found in adenoviral DNA and telomere regions of mammalian DNA [52–54].

Our results revealed a higher occurrence of the immunosuppressive TCAAGCTTGA motifs in C communities, which were particularly enriched in the *B*. *longum* population (*B*. *longum*–BFN-121-2). The TCAAGCTTGA motif inhibits DC activation, sustains Treg cell induction and suppresses the production of IFN-γ and IL-17 by T cells during inflammation [36]. Of interest, a Th1, Th17/Th22 dominant cytokine have been observed in chronic lesions of eczema [55]. This immunosuppressive motif may play a protective role in the development of eczema through inhibiting the development of Th1, Th17 cells, although this hypothesis awaits future experimental and clinical verifications. Our results further support the contribution of the specific *B*. *longum* population to the significantly higher levels of TCAAGCTTGA motifs found in C communities.

Commensal bacteria of the gut are also sources of multiple vitamins and cofactors [56]. In our study, C communities have higher abundance in genes in the subcategory of tetrapyrrole that includes pathways in porphyrins (heme), corrinoids (cobalamin) and chlorophyll metabolism. Selected porphyrins have shown to have anti-inflammatory effects [57, 58]. In contrast, coproporphyrin III, the most abundant extracellular porphyrin produced by human skin commensal *Propionibacterium spp* was shown to stimulate expression of the pro-inflammatory cytokine IL-8 by keratinocyte [59]. Cobalamin (vitamin B12) is an unusual vitamin. Only archaea and limited bacteria but not animals, plants and fungi are capable of de novo synthesis of cobalamin and its analogs (corrinoids). Though corrinoids are synthesized in a small set of gut microbes, more than 80% of human gut bacteria possess enzymes that require corrinoids as cofactor and many bacteria scavenge corrinoids from other microbes or the host’s diet. Hence the efficiency of using corrinoids in the gut microbial ecosystem may determine the gut microbial communities [60]. A relative abundance in genes related to tetrapyrrole biosynthesis in C communities suggests that microbes in healthy infants may produce more corrinoids and hence help the survival of ‘eczema protective’ bacteria *Bifidobacterium*, *Bacteroides* and *Lactobacillus*. These bacteria are, in general, incapable of de novo synthesis of corrinods and scavenge corrinoids from environment. Accordingly, further examination of specific classes of tetrapyrrole compounds derived from the neonatal gut microbiota (e.g., particularly *Veillonella*) will be of particular interest to assess the influence of the microbial metabolites on the pathogenesis of eczema.

## Conclusions

In summary, this study supports the notion that dysbiosis of neonatal gut microbiota is associated with subsequent development of eczema in infancy. Our findings suggest that the interplay between gut microbial DNA and human immune responses within a month of life influences the development of eczema. The study has several limitations. Foremost, would be the small sample size. Further, the group varied in terms of mode of delivery, breast feeding, ethnicity and family history of allergic disease. These findings, therefore, would require further validation with a larger study population. Our study also reports near-complete genome sequences of major *Bifidobacterium* populations derived from neonatal gut microbiota. The genomes of *Bifidobacterium* populations will be useful references on further investigation of selecting probiotic strains for the treatment of allergic diseases and understanding molecular mechanisms of probiotic benefits of the strains.

## Supporting information

S1 Table Characteristics of metagenomic datasets. All DNA samples were sequenced using both HiSeq 2500 and MiSeq V2 sequencers. Four samples (157, 176, 161, and 221) were further sequenced using a 454 FLX Titanium sequencer. The total size of combined metagenomic datasets from a sample ranged from 14 to 23 Gb. ^a^ Only contigs and genes longer than 300 bases were counted. ^b^ Chao richness and Shannon diversity indices were estimated based on 7,000 metagenomic reads that are overlapping and encoding V9 region of 16s rRNA genes (see details in Materials and Methods), which were randomly sampled using MOTHUR (See Reference 1 in S1 File). The Chao richness indices of C and E communities were 87 ± 32 and 91 ± 24, respectively. The Shannon diversity indices of C and E communities were 2.3 ± 0.5 and 2.1 ± 0.7, respectively.
(DOCX)

S2 Table Occurrence of five immune-stimulatory DNA motifs (See references 2 and 3 in S1 File) in C and E metagenomic datasets. ^a^ The value represent an average occurrence of the DNA motifs per 10^6^ metagenomic sequences. ^**b**^ *P* values were estimated by Mann-Whitney U test.
(DOCX)

S3 Table Genomic features of Bifidobacterium populations. ^a^ Source denotes the sample ID of a metagenomic dataset where the draft genome was recovered. ^**b**^ Only contigs and genes longer than 300 bases were counted. ^**c**^ Genome completeness was estimated using MaxBin^**3**^. ^**d**^ The genome sequences were deposited in GenBank under the accession numbers.
(DOCX)

S1 Fig Phylogenetic composition of microbial communities. Relative abundance of major phyla (see key) based on best match analysis of V9 regions of 16S rRNA gene sequences (left bar) and all metagenomic reads (right bar). ‘Others’ represents the combined fraction of the remaining minor phyla.
(TIF)

S2 Fig Rarefaction curves of metagenomic reads that encode the V9 region of 16S rRNA genes from each community. Curves represent the number (y-axis) of unique OTUs (defined at the 97% nucleotide sequence identity level) obtained per the number (x-axis) of sequences analyzed within each community (figure key). The rarefaction curves were produced using MOTHUR with 1000 permutations. Note that the number of OTUs recovered began to level off within each community.
(TIF)

S3 Fig Occurrence of the TCAGCTTGA motifs in complete genomes of the eight genera. The occurrence was estimated based on the complete genome sequences available in GenBank database and the number (n) of genomes analyzed is shown in parentheses. The bars represent average occurrence of the TCAGCTTGA motifs per 10^6^ bases. The error bars represent one standard deviation from the mean.
(TIF)

S4 Fig Phylogenetic tree of the six *Bifidobacterium* populations based on *polA* (DNA polymerase I) genes. The phylogenetic tree was built using MEGA 6.0 based on the maximum likelihood method with the Tamura-Nei model. Black circles represent *B*. sp. whose draft genomes were recovered in this study (S3 Table); the GI numbers of the remaining representative sequences from the GenBank database are provided in parentheses. The bootstrap support from 100 replicates is shown on the nodes of the tree.
(TIF)

S1 File Supporting information reference list. (DOCX)
