# Supplementary material for: Immune-modulatory genomic properties differentiate gut microbiota of infants with and without eczema
Source: PLoS One. 2017 Oct 19;12(10):e0184955. doi: 10.1371/journal.pone.0184955 (PMC5648123; doi:10.1371/journal.pone.0184955)
Supplement: S1 Table — All DNA samples were sequenced using both HiSeq 2500 and MiSeq V2 sequencers. Four samples (157, 176, 161, and 221) were further sequenced using a 454 FLX Titanium sequencer. The total size of combined metagenomic datasets from a sample ranged from 14 to 23 Gb. a Only contigs and genes longer than 300 bases were counted. b Chao richness and Shannon diversity indices were estimated based on 7,000 metagenomic reads that are overlapping and encoding V9 region of 16s rRNA genes (see details in Materials and Methods), which were randomly sampled using MOTHUR (See Reference 1 in S1 File). The Chao richness indices of C and E communities were 87 ± 32 and 91 ± 24, respectively. The Shannon diversity indices of C and E communities were 2.3 ± 0.5 and 2.1 ± 0.7, respectively. (DOCX) [file pone.0184955.s001.docx]

| Sample ID | Sample group | HiSeq 2500 (Mb) | MiSeq V2 (Mb) | 454 FLX Titanium (Mb) | Contig size (Mb) ^a^ | No. of genes ^a^ | G + C content (%) | Chao ^b^ | Shannon ^b^ |
| --- | --- | --- | --- | --- | --- | --- | --- | --- | --- |
| 157 | Control | 13,508 | 5,830 | 770 | 43 | 42,201 | 50 | 56 | 1.5 |
| 176 | Control | 15,247 | 6,580 | 780 | 66 | 65,693 | 49 | 96 | 2.7 |
| 192 | Control | 14,477 | 5,495 |  | 26 | 23,133 | 48 | 39 | 1.7 |
| 121 | Control | 12,423 | 5,586 |  | 96 | 86,284 | 48 | 116 | 2.6 |
| 165 | Control | 11,642 | 3,668 |  | 114 | 112,083 | 51 | 97 | 2.5 |
| 166 | Control | 11,060 | 3,327 |  | 77 | 76,793 | 48 | 118 | 2.4 |
| 161 | Eczema | 11,687 | 6,368 | 774 | 87 | 93,280 | 49 | 79 | 1.7 |
| 221 | Eczema | 10,082 | 5,265 | 715 | 36 | 36,754 | 52 | 61 | 1.6 |
| 128 | Eczema | 14,290 | 8,282 |  | 104 | 98,816 | 52 | 123 | 3.5 |
| 177 | Eczema | 13,772 | 5,438 |  | 84 | 85,973 | 50 | 116 | 2.5 |
| 141 | Eczema | 14,294 | 4,124 |  | 37 | 34,092 | 50 | 76 | 1.6 |
| 170 | Eczema | 11,958 | 4,077 |  | 67 | 65,220 | 49 | 90 | 1.9 |
