# Supplementary material for: Immune-modulatory genomic properties differentiate gut microbiota of infants with and without eczema
Source: PLoS One. 2017 Oct 19;12(10):e0184955. doi: 10.1371/journal.pone.0184955 (PMC5648123; doi:10.1371/journal.pone.0184955)
Supplement: S2 Table — a The value represent an average occurrence of the DNA motifs per 106 metagenomic sequences. b P values were estimated by Mann-Whitney U test. (DOCX) [file pone.0184955.s002.docx]

| Sequence | C^a^ | E^a^ | *P* value^b^ |
| --- | --- | --- | --- |
| AACGTT | 272 | 261 | 0.94 |
| AACGTTCG | 2.73 | 2.50 | 0.59 |
| AGCGCT | 227 | 223 | 1.0 |
| GACGTC | 153 | 162 | 1.0 |
| ATCGAT | 323 | 297 | 0.39 |
