# Supplementary material for: Immune-modulatory genomic properties differentiate gut microbiota of infants with and without eczema
Source: PLoS One. 2017 Oct 19;12(10):e0184955. doi: 10.1371/journal.pone.0184955 (PMC5648123; doi:10.1371/journal.pone.0184955)
Supplement: S3 Table — a Source denotes the sample ID of a metagenomic dataset where the draft genome was recovered. b Only contigs and genes longer than 300 bases were counted. c Genome completeness was estimated using MaxBin3. d The genome sequences were deposited in GenBank under the accession numbers. (DOCX) [file pone.0184955.s003.docx]

| Draft genomes | Source ^a^ | Total size (b) ^b^ | No. of contigs ^b^ | N50 of contigs ^b^ | No. of genes ^b^ | G + C content (%) | Completeness ^c^  (%) | Closest relative (Accession no.) | Genome identity (%) | Accession no. **^d^** |
| --- | --- | --- | --- | --- | --- | --- | --- | --- | --- | --- |
| *B. bifidum* BFY-141-5 | 141 | 2,105,224 | 44 | 104,216 | 1,500 | 62.9 | 96 | *B. bifidum* NCIMB (NZ_ABQP00000000) | 99.3 | LSUL00000000 |
| *B. bifidum* BFY-170-6 | 170 | 2,131,937 | 72 | 59,130 | 1,530 | 63.0 | 96 | *B. bifidum* NCIMB (NZ_ABQP00000000) | 99.3 | LSUI00000000 |
| *B. breve* BFY-141-3 | 141 | 2,347,263 | 53 | 87,501 | 1,729 | 58.8 | 95 | *B. breve* CECT_7263 (AFVV01000000) | 98.5 | LSUH00000000 |
| *B. dentium* FFN-176-7 | 176 | 2,611,332 | 47 | 98,823 | 1,957 | 58.5 | 96 | *B. dentium*_ATCC_27679 (NZ_GL405225) | 99.0 | LSUJ00000000 |
| *B. longum* BFN-121-2 | 121 | 1,872,563 | 234 | 14,757 | 1,336 | 60.3 | 94 | *B. longum* subsp._infantis_ATCC_55813 (NZ_ACHI00000000) | 99.3 | LSUK00000000 |
| *B. pseudocatenulatum* BFN-121-5 | 121 | 1,926,705 | 45 | 78,885 | 1,400 | 56.7 | 92 | *B. pseudocatenulatum* DSM_20438 (NZ_ABXX00000000) | 98.3 | LSUM00000000 |
