# Supplementary figures and images for: Immune-modulatory genomic properties differentiate gut microbiota of infants with and without eczema
Source: PLoS One. 2017 Oct 19;12(10):e0184955. doi: 10.1371/journal.pone.0184955 (PMC5648123; doi:10.1371/journal.pone.0184955)

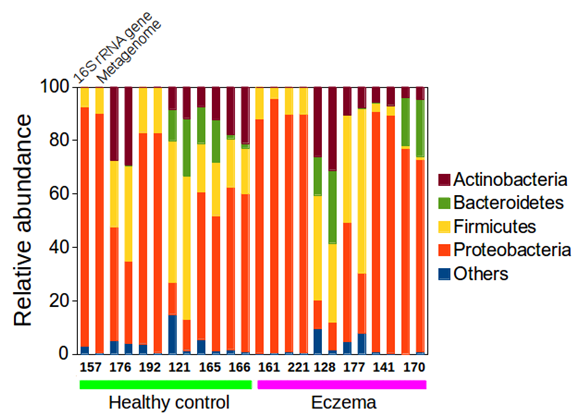

Supplement: S1 Fig — Relative abundance of major phyla (see key) based on best match analysis of V9 regions of 16S rRNA gene sequences (left bar) and all metagenomic reads (right bar). ‘Others’ represents the combined fraction of the remaining minor phyla. (TIF) [file pone.0184955.s004.tif]

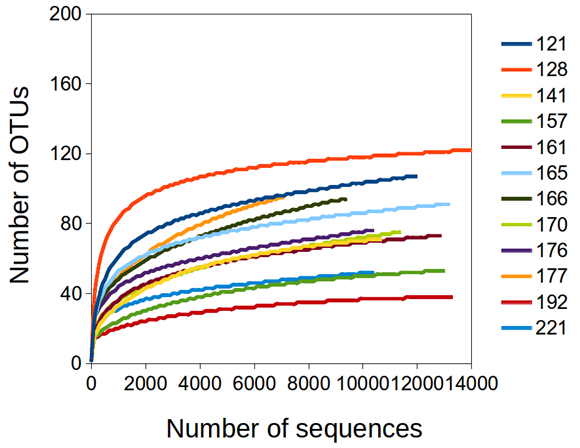

Supplement: S2 Fig — Curves represent the number (y-axis) of unique OTUs (defined at the 97% nucleotide sequence identity level) obtained per the number (x-axis) of sequences analyzed within each community (figure key). The rarefaction curves were produced using MOTHUR with 1000 permutations. Note that the number of OTUs recovered began to level off within each community. (TIF) [file pone.0184955.s005.tif]

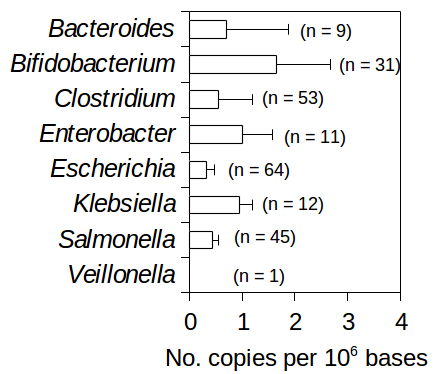

Supplement: S3 Fig — The occurrence was estimated based on the complete genome sequences available in GenBank database and the number (n) of genomes analyzed is shown in parentheses. The bars represent average occurrence of the TCAGCTTGA motifs per 106 bases. The error bars represent one standard deviation from the mean. (TIF) [file pone.0184955.s006.tif]

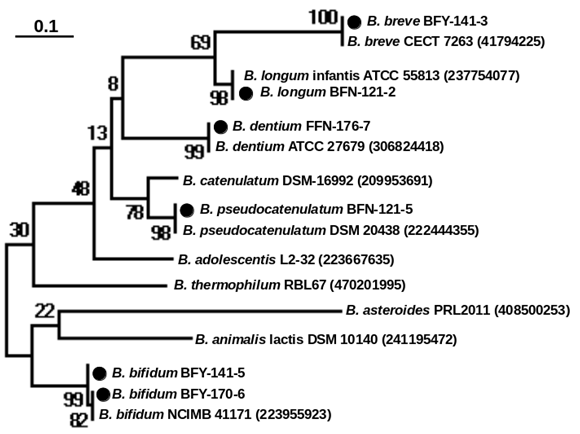

Supplement: S4 Fig — The phylogenetic tree was built using MEGA 6.0 based on the maximum likelihood method with the Tamura-Nei model. Black circles represent B. sp. whose draft genomes were recovered in this study (S3 Table); the GI numbers of the remaining representative sequences from the GenBank database are provided in parentheses. The bootstrap support from 100 replicates is shown on the nodes of the tree. (TIF) [file pone.0184955.s007.tif]
