# Supplementary material for: Immune-modulatory genomic properties differentiate gut microbiota of infants with and without eczema
Source: PLoS One. 2017 Oct 19;12(10):e0184955. doi: 10.1371/journal.pone.0184955 (PMC5648123; doi:10.1371/journal.pone.0184955)
Supplement: S1 File — (DOCX) [file pone.0184955.s008.docx]

**References for Supporting Information**

# [Schloss](http://www.ncbi.nlm.nih.gov/pubmed/?term=Schloss%20PD%5Bauth%5D) PD, [Westcott](http://www.ncbi.nlm.nih.gov/pubmed/?term=Westcott%20SL%5Bauth%5D) SL,  [Ryabin](http://www.ncbi.nlm.nih.gov/pubmed/?term=Ryabin%20T%5Bauth%5D) T, [Hall](http://www.ncbi.nlm.nih.gov/pubmed/?term=Hall%20JR%5Bauth%5D) TR, [Hartmann](http://www.ncbi.nlm.nih.gov/pubmed/?term=Hartmann%20M%5Bauth%5D) M, [Hollister](http://www.ncbi.nlm.nih.gov/pubmed/?term=Hollister%20EB%5Bauth%5D) EB, et al. Introducing mothur: Open-Source, Platform-Independent, Community-Supported Software for Describing and Comparing Microbial Communities. Appl Environ Microbiol 2009; 75:7537-41

1. Van Uden J and Raz E. Immunostimulatory DNA and applications to allergic disease. J Allergy Clin Immunol 1999; 104:902-10
2. Bouladoux N, Hall JA, Grainger JR, dos Santos LM, Kann MG, Nagarajan V, et al. Regulatory role of suppressive motifs from commensal DNA. Mucosal Immunol 2012; 5:623-34
